# Commentary on Acquired Factor XI Deficiency during SARS-CoV-2 Infection: Not Only Thrombosis

**DOI:** 10.1055/s-0040-1716839

**Published:** 2020-09-13

**Authors:** Maria E. de la Morena-Barrio

**Affiliations:** 1 Servicio de Hematología y Oncología Médica, Hospital Universitario Morales Meseguer, Centro Regional de Hemodonación, Universidad de Murcia, IMIB-Arrixaca, CIBERER, Murcia, Spain

Coronavirus disease (COVID-19) is threatened our societies and the need for personalized medicine and efficient drugs are compulsory. COVID-19 can lead to systemic coagulation activation and thrombotic complications. Excessive cytokine release and activation of coagulation are key drivers of COVID-19 pneumonia and associated mortality. Contact activation has been linked to pathologic upregulation of both inflammatory mediators and coagulation. Congenital factor XI (FXI) deficiency is more frequent than previously expected, as it has been demonstrated in numerous reports and all over the world. Although it was first described prevalent in exclusively etnias (Ashkenazy or Iraqi-Jewish), it seems to be misdiagnosed and not so exclusive, with a potential high prevalence at least in heterozygosis, also in Europe. 1 Moreover, acquired deficiency can occur with the presence of inhibitors or antibodies such as antiphospholipid as β2-glycoprotein I. 2 Whether or not COVID-19 contributing to the appearance of antiphospholipid antibodies, is still unclear and requires further studies.

**Fig. 1:**
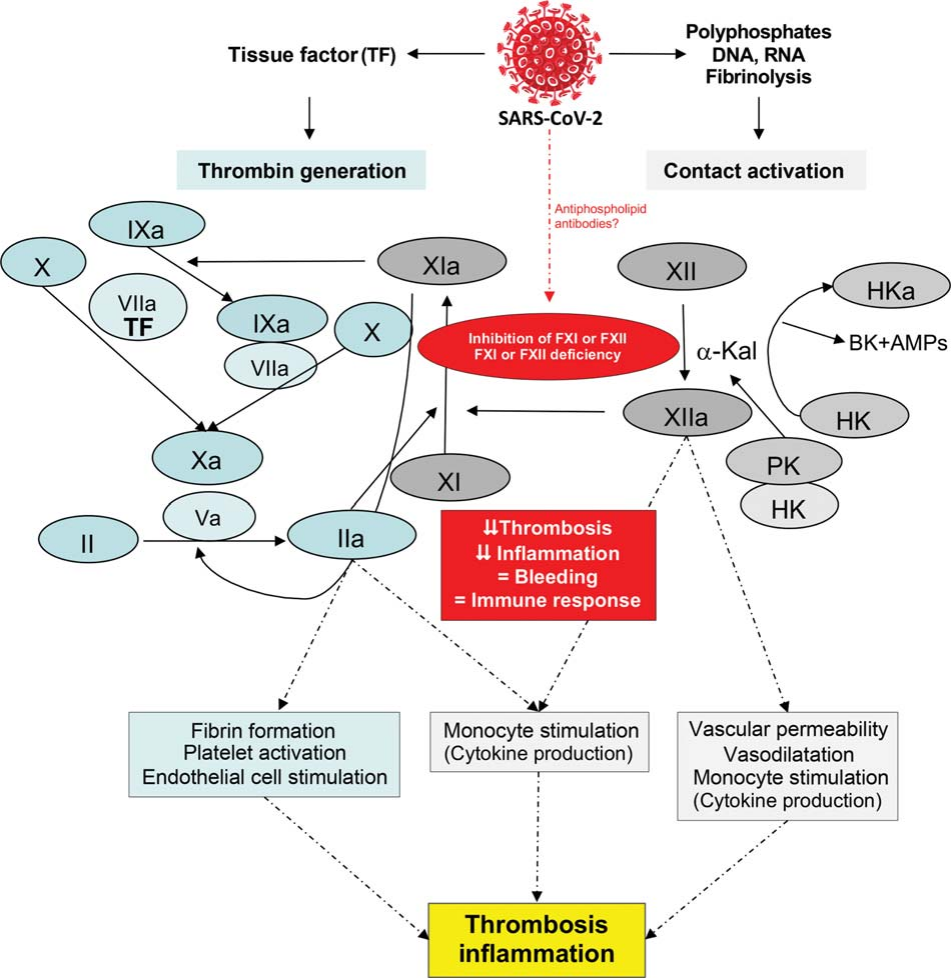
Schematic representation of the potential role of FXI and FXII in the thrombotic and inflammation complications induced by SARsS-CoV-2 infection. The antithrombotic and anti-inflammatory capacity of the inhibition of contact pathway and FXI and FXII deficiency could be a therapeutic option in this disease with low risk of bleeding. Whether SARS-CoV-2 infection could produce antiphospholipid antibodies able to cause an acquired FXI deficiency with protective role remains to be clarified. F, factor; SARS-CoV-2, severe acute respiratory syndrome-coronavirus-2.

FXI is a pleiotropic molecule whose role is extended from coagulation to inflammation. The main role in the clot cascade is to amplify the thrombin activation. However, its deficiency in humans or in animal models does not produce bleeding even under risk situations, but importantly, it reduces the risk of thrombosis. Indeed, pharmacological or molecular inhibition of FXI has been shown to prevent thrombosis without increasing the bleeding risk. 3 Besides, FXI is also involved in the inflammatory system as part of the contact activation pathway with FXII. Therapeutic drugs inhibiting this molecule have shown to prevent coagulopathy, systemic inflammatory response, and also mortality in experimental sepsis and without immune compromise ( Fig. 1 ).

For that reason, the description and characterization of COVID-19 patients with congenital or acquired FXI deficiency, as the one presented by Andreani et al in this issue, 4 could help to understand the disease and to identify new targets in this disease. They can also contribute to show new evidences supporting a personalized medicine. Indeed, if FXI deficiency is modulating the risk of thrombosis in COVID-19, we can assume that the coagulation is some way through the action of this coagulation factor (and probably the contact pathway), making very attractive to consider drugs targeting FXI to prevent thrombotic complication. The potential effect of these treatments in the respiratory distress mediated to cytokine release might also be evaluated. However, this is only one case report and further studies of FXI-deficient patients with COVID-19 would be of great value to support this hypothesis.
